# Supplementary material for: Establishing Human and Canine Xenograft Murine Osteosarcoma Models for Application of Focused Ultrasound Ablation
Source: Biomedicines. 2025 Aug 30;13(9):2122. doi: 10.3390/biomedicines13092122 (PMC12467533; doi:10.3390/biomedicines13092122)
Supplement: Supplementary file 1 [file biomedicines-13-02122-s001.zip › biomedicines-3822927-supplementary.pdf]

Supplemental Table S1: Human murine xenograft death timepoints. The number of mice in each group at each timepoint which a death of a tumor-bearing study animal occurred is reported in either the untreated or histotripsy ablated (histotripsy) groups.

Model	Days Post Tumor Cell Injection	Group	Number of Mice/Group
Human	22	Untreated	5
		Histotripsy	*6*
	23	Untreated	5
		Histotripsy	*6*
	24	Untreated	5
		Histotripsy	*6*
	25	Untreated	3
		Histotripsy	*6*
	26	Untreated	2
		Histotripsy	*5*
	27	Untreated	0
		Histotripsy	*4*
	28	Untreated	0
		Histotripsy	*3*
	29	Untreated	0
		Histotripsy	*0*

Supplemental Table S2: Canine murine xenograft death timepoints. The number of mice in each group at each timepoint which a death of a tumor-bearing study animal occurred (day post tumor cell injection) is reported in either the untreated or histotripsy ablated (histotripsy) groups.

Xenograft Model	Days Post Tumor Cell Injection	Group	Number of Mice/Group
Canine	57	Untreated	2
		Histotripsy	*3*
	63	Untreated	0
		Histotripsy	*3*
	70	Untreated	0
		Histotripsy	*1*
	90	Untreated	0
		Histotripsy	*0*
